# Young people’s awareness of the timing and placement of gambling advertising on traditional and social media platforms: a study of 11–16-year-olds in Australia

**DOI:** 10.1186/s12954-018-0254-6

**Published:** 2018-10-19

**Authors:** Samantha L. Thomas, Amy Bestman, Hannah Pitt, Rebecca Cassidy, Simone McCarthy, Christian Nyemcsok, Sean Cowlishaw, Mike Daube

**Affiliations:** 1 Centre for Population Health Research, School of Health and Social Development, Faculty of Health, Deakin University, Geelong, Australia; 2 Department of Anthropology, Goldsmiths, University of London, London, UK; 3 Department of Psychiatry, The University of Melbourne, Melbourne, Australia; 4 Population Health Science, Bristol Medical School, University of Bristol, Bristol, UK; 5 Faculty of Health Sciences, Curtin University, Bentley, Australia

**Keywords:** Gambling, Advertising, Children, Social media, Television, Sport

## Abstract

**Background:**

Research has demonstrated that the promotion of gambling, particularly within sport, may have a significant impact on positively shaping young people’s attitudes towards gambling. While some governments have implemented restrictions to limit young people’s exposure to gambling advertising, few studies have investigated where young people recall seeing gambling advertising, and whether they perceive that advertising restrictions have gone far enough in reducing exposure to these promotions.

**Method:**

Mixed methods, interviewer-assisted surveys were conducted with *n* = 111 young people aged 11–16 years, who were self-reported fans of basketball in Victoria, Australia. Interviews were conducted at basketball stadiums between May and July 2018. The study assessed media viewing patterns; recall and awareness of the timing, placement, and content of gambling advertising; the impact of gambling advertising restrictions; and attitudes towards sporting organisations’ roles in the promotion of gambling.

**Results:**

The majority of young people recalled seeing gambling advertising on television (*n* = 101, 91.0%), with most recalling advertising within sporting matches or games (*n* = 79, 71.2%). Most young people recalled seeing gambling advertising in the early evening before 8:30 pm (*n* = 75, 67.6%). Just over half of young people described seeing gambling advertisements on social media (*n* = 61, 55.0%), and over a third (*n* = 40, 36.0%) recalled gambling advertising on YouTube, predominantly before watching sporting or gaming videos. The majority stated that they continued to watch sport after 8:30 pm (*n* = 93, 83.7%), which is when restrictions on advertising in live sport in Australia end. The majority (*n* = 88, 79.3%) stated that there were too many gambling advertisements in sport. Three quarters believed that sporting codes should do more to prevent young people from being exposed to advertising for gambling in sport (*n* = 84, 75.7%).

**Conclusions:**

There is now a clear body evidence that current regulatory systems for gambling advertising are ineffective, with further restrictions urgently needed across a range of media channels to prevent exposure to promotions that may encourage young people’s interest and involvement in gambling.

## Background

### Young people and gambling-related harm

The impact of gambling on children and young people (subsequently referred to as young people) has emerged as an important public health issue in the last decade [1–3]. Researchers have demonstrated that approximately 60–80% of young people engage in formal or informal gambling prior to the legal age in various jurisdictions and are vulnerable to harmful and problem gambling [4–7]. For example, a survey conducted by the Gambling Commission in the United Kingdom (UK) estimated that around 0.9% of 11- to 15-year-olds were problem gamblers (equating to 31,000 young people) and a further 1.3% were “at-risk” of problem gambling and currently exhibiting at least some problematic behaviours or harms (equating to an additional 45,000 young people) [8]. Annual estimates of gambling in Great Britain have indicated large increases in participation among young people aged 16–24 years, with 38% participating in gambling in 2016, up to 5% from the previous year [9]. In Finland, a survey of 12- to 15-year-olds found that 3.0% identified as problem gamblers and a further 4.9% were at risk [10]. In a Swedish longitudinal study, incidence of problem gambling in 16- to 24-year-olds was over double the rate for adults aged 25–44 years—2.26% compared to 0.81% for adults [11]. In Australia, research suggests that 1 in 25 young people have experienced a problem with gambling in the previous year [12], with one Australian study finding that 16% of young people under 25 were classified as at-risk, and 5% were classified as problem gamblers [13].

Researchers have explored a range of individual [14, 15], peer [16], and family [17–19] factors that may influence and shape young people’s attitudes and engagement with gambling. However, it is the alignment of online sports betting companies with professional sporting teams and codes [2], the saturation of advertising on television [20, 21], and research demonstrating the positive attitudes of young sports fans towards gambling [22–24] that have stimulated the most public debate about the normalisation of gambling for young people.

### The impact of gambling advertising on young people

In the last 5 years, a range of studies, mostly from Australia, have examined the alignment across gambling companies and sporting codes and impacts on gambling attitudes and consumption intentions of young people [2, 3, 18, 23, 25, 26]. These studies have clustered around three key themes.

The first examines young people’s recall and awareness of sports betting brands and demonstrates that they can recall the names of gambling companies, describe the distinct marketing strategies aligned with these companies, and associate gambling company sponsors with relevant sports teams [2, 3, 23]. For example, Thomas and colleagues [2] found that over 75% of 8- to 16-year-olds could recall the brand name of at least one sports betting company.

The second examines young people’s interpretations of gambling from appeal and communication strategies within advertisements [3, 23]. These studies have demonstrated that specific types of appeal strategies within sports betting promotions, such as cash back deals or offers, have an impact on young people by reducing the perceived risk associated with engaging in gambling [3, 18]. Other studies have demonstrated that celebrity endorsement of sports betting companies may increase perceptions of trust and that gambling is an essential part of a sports fan’s identity [25]. Most recently, researchers have demonstrated that patterns of observational learning may result from marketing, whereby the technical aspects of betting, such as opening accounts and betting via mobile phones, are modelled for young people [3].

The third examines young people’s gambling intentions, although few of these provide significant detail about exactly *why* young people may be motivated to gamble [18, 26, 27]. For example, Derevensky and colleagues [27] found that 40% of young people stated they wanted to try gambling after seeing gambling advertisements. Other studies have demonstrated that young people who watch sport are more likely to indicate a desire to try gambling as adults [26]. Pitt et al. [18] found in a qualitative study that over 60% of young people wanted to try gambling (currently or in the future) and 35% selected sports betting as the gambling product they would most like to try. The study identified four key factors which influenced the gambling intentions of young people: the alignment of gambling with culturally valued events such as sport; having perceived expert knowledge and understanding of sport; their awareness of gambling advertising and promotions within sport; and the attitudes and behaviours of parents and peers towards gambling.

Despite this work, there are still gaps in understanding of the impact of gambling promotional strategies on young people, and the most effective public policy strategies to respond to these. First, research has been substantially skewed towards boys who are fans of major sporting codes, with limited research on whether girls are also influenced by gambling advertising [18, 28]. This is important given recent research suggesting that women are a growing market for gambling companies and are increasingly engaging in online forms of gambling [29]. There is also very limited information about the specific media channels that are used to promote gambling and how young people may be exposed to gambling advertising via different channels. For example, while young people are significant consumers of information on social and digital media platforms [30], there has been very limited research on the nature and extent of gambling advertising on these platforms [31]. Given research demonstrating that alcohol and tobacco brands have used social media platforms to market their products, and that young people may be exposed regularly to these promotions [32, 33], it is also important to understand the extent and nature of gambling advertising on these media platforms. Finally, measures associated with protecting young people from gambling promotions are predominantly related to the timing of advertisements within young people’s viewing hours [34]. However, very few of these are based on evidence about when and where young people see gambling advertising in their general media viewing and everyday environments [23], and whether regulations based on a “watershed” for televised commercial break advertising have any impact on young people’s overall exposure to gambling advertising.

### Regulatory responses

There have been various regulatory responses to the promotion of gambling within sport across the world, with Italy, the UK, and Australia providing examples of diverse policy responses. For example, Italy has committed to implementing a blanket ban on all gambling advertising across all media platforms [35], an intervention condemned by Maarten Haijer, secretary general of the European Gaming and Betting Association, as impractical and “counterproductive” [36]. Should these rules come into force as planned in January 2019, sports clubs will also be banned from carrying sponsorship promotions provided by the industry. At present, more than half of the teams in Italy’s top football league have sponsorship agreements with gambling providers [37].

In the UK, the Gambling Act (2005) legalised gambling advertising on all media. However, the Industry Code for Socially Responsible Gambling, which also came into force in 2007, imposed a 9 pm television watershed for all gambling products except bingo, and sports betting around televised sports events [38]. Despite considerable pressure from campaigners and parliamentarians [39], the UK Government has resisted calls to ban gambling advertising before the watershed. In 2016, the UK Government launched a review of social responsibility measures and advertising. Their response, published in May 2018, made no changes to existing rules on advertising. Rather, it welcomed a major multi-million-pound advertising campaign led by GambleAware, around responsible gambling, and promised new research commissioned by GambleAware which would explore “the effects of gambling advertising and marketing on children, and vulnerable groups” [40].^1^

Relative to these European countries, Australia has taken what may be described as a “middle ground” approach to the promotion of gambling in sport. While there have been state-based initiatives aimed at restricting gambling advertising in public spaces such as near schools and at major public transport stations [42], regulations were introduced at a national level on 30 March 2018 that restricted the advertising of gambling during live sports broadcasts between 5:00 am and 8:30 pm [43]. In August 2018, regulations were also announced to ban gambling advertising during the online streaming of live sport between 5:00 am and 8:30 pm [44]. While the introduction of these rules was designed to “limit the exposure of child audiences to gambling ads and promotion of odds during live sporting events” [43], the regulations contain a number of loopholes and exemptions which have important implications for young people’s continued exposure to gambling advertising. First, the regulations do not apply to subscription television channels with a “low” audience share [43]. This means that channels on subscriptions services such as ESPN which broadcast sports including the American National Basketball Association (NBA) can continue to broadcast gambling advertising between 5:00 am and 8:30 pm [45]. Second, the 8:30 pm cut-off does not appear to be based on analyses of the media viewing patterns of young people, and specifically young people’s viewing patterns associated with sport. For example, in a submission to the Inquiry into the Communications Legislation Amendment (Online Content Services and Other Measures) Bill 2017, UNICEF Australia (a multilateral organisation which aims to protect the rights of children) highlighted that while the Regulation Impact Statement associated with the Australian governments proposed advertising restrictions stated: “[t]here is less concern where these events are broadcast after 8:30pm as children are less likely to be viewing at this time...” no evidence was provided to support this statement ([46], p. 8). In their submission to the Inquiry, UNICEF Australia challenged the proposition that 8:30 pm was an appropriate cut-off time, providing evidence that young people watched media content well beyond 8:30 pm, and increasingly engaged in media viewing via online digital platforms [46]. Third, the regulations only apply to live sport, with a starting point for restrictions from 5 min prior to the match. This means that gambling advertising (including sponsor announcements) may be played in sports commentary in the lead-up to sports matches, and in replays of popular matches. Fourth, there are still exemptions for gambling advertising within sports and current affairs (news) programs, such as sports magazine or commentary programs, prior to 8:30 pm. Fifth, restrictions do not apply to incidental advertising including hoardings at stadiums or logos on a player’s or official’s uniform, which may still be highly visible throughout live matches and have implicit recall for young people [2, 47]. Finally, the regulations do not apply to gambling advertising on social media platforms, including promoted content on YouTube, Instagram, and Snapchat, which are all used by young people.

While the regulations developed by the Australian government were a step in the right direction, these gaps may mean that young people are still exposed to significant gambling advertising during their general and sports-related media viewing. The following study aimed to enhance understanding of young people’s exposure and awareness of gambling advertising across different media platforms since the March 2018 implementation of new gambling advertising restrictions in Australia. The analyses presented in this paper were guided by four research questions:

1. To what extent do young people report seeing gambling advertising on different media channels?
2. What do young people recall about the timing and placement of gambling advertising via different media channels?
3. Have young people noticed any changes in gambling advertising since regulatory restrictions were introduced by the Australian government?
4. How do young people perceive the responsibility of sporting organisations in relation to gambling advertising?

## Methods

### Approach

This study was part of a broader mixed methods, interviewer-administered survey of gambling attitudes and advertising recall of 11- to 16-years-olds in Victoria, Australia. The study specifically focused on young people who were fans of basketball for two reasons. First, the vast majority of research in Australia has focused on young people who are fans of two major sporting codes, the Australian Football League (AFL) and the National Rugby League [2, 18, 23], so it was considered worthwhile to investigate the attitudes of young people engaged with other sports. Second, given the “pay per view” access of this sporting code in Australia, we hypothesised that young people who were fans of the NBA but did not have access to paid media platforms might seek content via free social media platforms. This was important in understanding whether young people were exposed to gambling advertising when viewing sports-related content on social media.

Greater than low risk ethical approval was received from the Deakin University Human Research Ethics Committee (2018-087) given that this project focused on young people, and given that gambling in Australia is illegal for under 18-year-olds.

### Sampling and recruitment

The study recruited 11- to 16-year-olds who were self-reported fans of basketball. The “fan” criterion was included given research suggesting that young people who are fans of sport may be more engaged in media viewing of sport, may be more exposed to gambling advertising, and may be most likely to indicate that they will gamble when they are older [18, 23]. The 11- to 16-year-old age range was chosen as research indicates that from the age of about 11, young people become more aware of the persuasive intent of marketing brands and communications [48, 49]. While the study predominantly utilised a convenience approach to sampling, purposive sampling was also used to ensure the diversity of the sample according to gender, socio-economic status, and age. This was important given that previous studies have predominantly focused on boys from higher socio-economic backgrounds [3, 50].

Young people were recruited between May and July 2018 at three local community basketball stadiums in Victoria, Australia. This time period was chosen as it was approximately the same time as the 2018 NBA playoffs and finals. Up to five trained researchers attended stadiums to recruit participants and collect data on 7 days. ST attended all 7 days of data collection to talk to parents, answer questions, and be on hand in case problems emerged. Parents and young people were approached and provided with written and verbal information about the study and were given an opportunity to ask questions prior to participation. There was a requirement from the University Ethics Committee for parents/carers to provide written consent for all young people, and for young people to provide verbal consent prior to participation. This requirement led to some difficulty in recruiting 15- and 16-year-olds, who often attended games without a parent. Young people received a non-branded drink bottle as a token of appreciation for participation.^2^

### Data collection

Data collection took place in a quiet part of the stadium. A one-on-one interviewer-assisted survey was completed individually with the young person using an iPad or iPhone 8 plus. Data were collected using the Qualtrics offline application, with questions asked and data entered by the interviewer. Qualitative data were recorded verbatim by the interviewer. Young people were given prompts throughout the survey that there were no right or wrong answers. Parents were able to observe the interview, although most chose not to. Surveys took between 10 and 15 min to complete and were piloted with 10 young people to check for comprehension. Minor adjustments were made to the wording of some questions which were difficult for young people to understand before proceeding with the full study.

The survey was divided into five sections which included a range of questions relating to socio-demographic characteristics, self-reported fan engagement, media viewing patterns, recall and awareness of gambling advertising, future gambling consumption intentions, awareness of gambling regulations, and suggestions for preventing gambling harm in young people. This study reports quantitative and qualitative data from four of these sections.

#### Socio-demographic and sporting characteristics

Age, gender, suburb of residence, and participation in sports.

#### Media viewing of sport

To understand media viewing of basketball, we included a check-list of items indicating types of basketball codes watched (e.g. NBA, NBL, Australian and American women’s basketball leagues, and American College Basketball); how often they watched these codes (daily, 4–6 times per week, 2–3 times per week, once a week, once a month, less than once a month); and the media platforms through which they viewed basketball (e.g. free to air television, subscription television, YouTube, websites). To understand broader engagement with sports on social media platforms, young people were asked which social media platforms they used to follow basketball players and teams.

#### Recall of the timing and placement of gambling advertising

Young people were asked a range of open and closed questions relating to whether they had seen gambling advertising on traditional and social media platforms. They were asked specifically where they recalled seeing this advertising—recorded via a check-list of eight traditional, online, and venue-based advertising channels. They were then asked qualitatively to describe in which specific television programs they had recalled seeing advertising. Finally, they were asked about times of day in which they had seen gambling advertising on traditional television media platforms (from a list of morning, afternoon, early evening before 8:30 pm, late evening after 8:30 pm).

#### Impact of gambling advertising restrictions

A number of questions explored the impact of the recently introduced gambling advertising restrictions. These included a yes/no question related to whether they continued to watch television after 8:30 pm at night, with a prompt regarding whether they would continue to watch a sporting match if aired after 8:30 pm. Young people were then asked questions relating to their perceptions about the amount of gambling advertisements in sport, for example, “what do you think about the amount of gambling ads in sport?” If young people did not immediately answer they were then given the following prompt, “do you think there is too much, too little, or about the right amount of ads?” They were then asked “do you think that sporting codes should do more to stop young people from being exposed to ads for gambling in sport?” which was coded as a yes/no response. Finally, young people were asked in an open-ended question whether they had a message for the sporting codes.

Given the mixed methods nature of the study, qualitative data were analysed as the study progressed, and data collection ceased when no new themes emerged from the qualitative responses.

### Data analysis

Data were uploaded to Statistical Package for the Social Sciences (SPSS) from Qualtrics for analysis. All data was checked and cleaned by the second and third authors before being coded. The main adjustments were minor typographical and wording issues associated with input of the qualitative data. Quantitative data were analysed using descriptive statistics in SPSS. Socio-Economic Indexes for Areas (SEIFA) status was determined using suburb level data. Suburbs were assigned scores according to SEIFA State Suburb Index of Relative Socio-economic Disadvantage [51]. These scores were grouped into low (deciles 1–3), middle (deciles 4–7), and high (deciles 8–10).

Significant differences according to gender and age were identified using *χ*^2^ tests of independence. To analyse data associated with age, we split the sample into two groups, young people aged 11–12 years (*n* = 55) and 13–16 years (*n* = 56). This was done for two reasons, first that it divided the data into two even groups and second because 13 is the age at which young people are permitted to open social media accounts in Australia [52].

Qualitative responses were transferred to data management software QSR NVivo 11 and were thematically analysed [53]. In this paper, qualitative responses are primarily used to illustrate or provide depth of information to complement quantitative data. Qualitative responses were read and re-read to understand the key concepts and themes emerging from the data. Regular meetings were held between the co-authors to discuss emergent themes.

## Results

### Socio-demographic characteristics

The socio-demographic characteristics of the sample are presented in Table 1. A total of 111 young people participated in the study, with a mean age of 12.9 years (SD 1.5). Approximately half were aged 11 or 12 years (*n* = 55, 49.5%), approximately a third aged 13 or 14 years (*n* = 39, 35.1%), and 15.3% were aged 15 or 16 years (*n* = 17). From this point forward, young people will be grouped into two age groups, 11–12 years (n = 55) and 13–16 years (*n* = 56). The sample was skewed towards boys (*n* = 66, 59.5%), but was roughly representative of basketball participation rates by gender in the state of Victoria [54], and a higher proportion of girls than in previous studies [2, 3]. The majority of young people lived in suburbs with SEIFA codes between four and seven (*n* = 64, 57.7%).

**Table 1:** Socio-demographic characteristics

Characteristic	*n* (%)
Age	
11–12 years	55 (49.5)
13–14 years	39 (35.1)
15–16 years	17 (15.3)
Gender	
Male	66 (59.5)
Female	45 (40.5)
SEIFA	
Low (scores 1–3)	18 (16.2)
Middle (scores 4–7)	64 (57.7)
High (scores 8–10)	28 (25.2)
Not provided	1 (0.9)

### Sports engagement characteristics

The sports engagement characteristics of the sample are provided in Table 2. The majority of participants played basketball for a domestic or representative team (*n* = 108, 97.3%), with a quarter of young people also playing AFL for a club-based team (*n* = 26, 23.4%). Sixteen (14.4%) reported participating in another sport such as soccer, hockey, netball, or dancing.

**Table 2:** Sports engagement and media viewing characteristics

	Gender		Age		Total (*n* = 111)
	Male (*n* = 66)	Female (*n* = 45)	11–12 (*n* = 55)	13–16 (*n* = 56)	
Sport played, *n* (%)					
Basketball	66 (100.0)*	42 (93.3)*	53 (96.4)	55 (98.2)	108 (97.3)
AFL	20 (30.3)*	6 (13.3)*	14 (25.5)	12 (21.4)	26 (23.4)
Other	5 (7.6)*	11 (24.4)*	14 (25.5)^^^	2 (3.6) ^^^	16 (14.4)
Type of basketball watched, *n* (%)					
Any basketball	65 (98.5)*	38 (84.4)*	50 (90.9)	53 (94.6)	103 (92.8)
NBA	56 (84.8)*	31 (68.9)*	43 (78.2)	44 (78.6)	87 (78.4)
NBL	50 (75.8)*	24 (53.3)*	36 (65.5)	38 (67.9)	74 (66.7)
College	14 (21.2)*	3 (6.7)*	0^^^	17 (30.4) ^^^	17 (15.3)
Other	14 (21.2)	14 (31.1)	11 (20.0)	17 (30.4)^^^	28 (25.2)
Basketball viewing (Media and live platforms), *n* (%)					
Subscription TV	30 (45.5)	17 (37.8)	25 (45.5)	22 (39.3)	47 (42.3)
Free to air TV	18 (27.3)	18 (40.0)	21 (38.2)	15 (26.8)	36 (32.4)
YouTube	17 (25.8)	9 (20.0)	12 (21.8)	14 (25.0)	26 (23.4)
Websites	16 (24.2)*	1 (2.2)*	4 (7.3) ^^^	13 (23.2) ^^^	17 (15.3)
NBA league^+^	9 (13.6)*	1 (2.2)*	4 (7.3)	6 (10.7)	10 (9.0)
Go to game	5 (7.6)	3 (6.7)	3 (5.5)	5 (8.9)	8 (7.2)
Other	6 (9.1)	2 (4.4)	4 (7.3)	4 (7.1)	8 (7.2)
Social media used to follow basketball, *n* (%)					
Total	40 (60.6)	23 (51.1)	24 (43.6) ^^^	39 (69.6) ^^^	63 (56.8)
Instagram	36 (54.5)	22 (48.9)	19 (34.5) ^^^	39 (69.6) ^^^	58 (52.3)
YouTube	21 (31.8)*	3 (6.7)*	9 (16.4)	15 (26.8)	24 (21.6)
Snapchat	19 (28.8)*	5 (11.1)*	6 (10.9) ^^^	18 (32.1)^^^	24 (21.6)
Website	6 (9.1)	1 (2.2)	3 (5.5)	4 (7.1)	7 (6.3)
Facebook	2 (3.0)	3 (6.7)	1 (1.8)	4 (7.1)	5 (4.5)
Twitter	1 (1.5)	0	0	1 (1.8)	1 (0.9)
Other	5 (7.6)	1 (2.2)	1 (1.8)	5 (8.9)	6 (5.4)

*n* = Number of participants, % = Column percentages

*Significance between genders at 0.05

^^^Significance between age groups at 0.05

^+^NBA league includes NBA league pass or streaming direct from NBA apps

The majority reported watching professional basketball games in the last six months (*n* = 103, 92.8%). Young people watched basketball regularly, with almost two thirds stating that they watched professional basketball at least once a week (*n* = 71, 64.0%), and approximately one in ten having watched daily (*n* = 14, 12.6%). Over half reported watching more than one type of professional basketball in the last six months (*n* = 66, 59.5%), with the NBA watched most (*n* = 87, 78.4%). Boys were significantly more likely to watch all forms of basketball except “other” as compared to girls [NBA—*χ*^2^ = 4.0, *p* = .045; NBL—*χ*^2^ = 6.1, *p* = .014; College—*χ*^2^ = 4.4, *p* = .037]. A third of young people stated that their media viewing of basketball had increased since the NBA playoffs (*n* = 20, 36.4%).

Young people watched basketball via a range of media platforms, including free to air and subscription television (*n* = 78, 70.2%), via YouTube (*n* = 26, 23.4%) or other websites (*n* = 17, 15.3%). While just over half of all young people said they followed basketball players or teams on social media (*n* = 63, 56.8%), 13- to 16-year-olds were significantly more likely to utilise social media for basketball engagement as compared to 11- to 12-year-olds [*χ*^2^ = 7.7, *p* = .006]. The top three social media platforms used to follow basketball players or teams were Instagram (*n* = 58, 52.3%), YouTube (*n* = 24, 21.6%), and Snapchat (*n* = 24, 21.6%). Those aged 13- to 16-years were significantly more likely to use Instagram [*χ*^2^ = 13.7, *p* = .00], and Snapchat [*χ*^2^ = 7.4, *p* = .007] compared to 11- and 12-year-olds. Boys were more likely to use YouTube [*χ*^2^ = 10.0, *p* = .002] and Snapchat [*χ*^2^ = 4.9, *p* = .026] as compared to girls.

### Recall of the placement and timing of gambling advertising on media channels

Young people’s recall of the placement and timing of gambling advertising on media channels is provided in Table 3. The majority of young people recalled seeing gambling advertising on media platforms (*n* = 107, 96.4%). There were no discernible differences according to age or gender for media type, with the exception of Snapchat, where 13- to 16-year-olds were significantly more likely to state they had seen gambling advertising, relative to 11- to 12-year-olds [*χ*^2^ = 9.5, *p* = .009].

**Table 3:** Recall of the placement and timing of gambling advertising on media channels

	Gender		Age		Total (*n* = 111)
	Male (*n* = 66)	Female (*n* = 45)	11–12 (*n* = 55)	13–16 (*n* = 56)	
Recall of the placement of gambling advertising, *n* (%)					
Television	58 (87.9)	43 (95.6)	50 (90.9)	51 (91.1)	101 (91.0)
YouTube	25 (37.9)	15 (33.3)	17 (30.9)	23 (41.1)	40 (36.0)
Instagram	10 (15.2)	4 (8.9)	4 (7.3)	10 (17.9)	14 (12.6)
Website	8 (12.1)	2 (4.4)	4 (7.3)	6 (10.7)	10 (9.0)
Snapchat	4 (6.1)	1 (2.2)	0^^^	5 (8.9) ^^^	5 (4.5)
At game	3 (4.5)	2 (4.4)	2 (3.6)	3 (5.4)	5 (4.5)
Facebook	3 (4.5)	1 (2.2)	1 (1.8)	3 (5.4)	4 (3.6)
A team app	2 (3.0)	0	2 (3.6)	0	2 (1.8)
Other	3 (4.5)	3 (6.7)	3 (5.5)	3 (5.4)	6 (5.4)
Time of day recalled seeing gambling advertising (in television programs), *n* (%)					
Morning	9 (13.6)*	1 (2.2)*	5 (9.1)	5 (8.9)	10 (9.0)
Afternoon	15 (22.7)	11 (24.4)	16 (29.1)	10 (17.9)	26 (23.4)
Early evening (before 8.30 pm)	42 (63.6)	33 (73.3)	36 (65.5)	39 (69.6)	75 (67.6)
Evening (after 8.30 pm)	28 (42.4)	22 (48.9)	22 (40.0)	28 (50.0)	50 (45.0)

*n* = Number of participants, % = Column percentages

*Significance between genders at 0.05

^^^Significance between age groups at 0.05

The majority of young people recalled seeing gambling advertising on television (*n* = 101, 91.0%). When asked open-ended questions about types of programs in which they had seen gambling advertising, most reported sporting matches or games (*n* = 79, 71.2%), including during AFL games (*n* = 55, 49.5%). Eleven commented that they had seen gambling advertising during basketball games. During sporting matches, young people described seeing advertising before games started, during advertising breaks, between games, or on hoardings around the ground or on the court. Young people also reported seeing gambling advertising in a range of other television programs, including entertainment or reality shows (*n* = 28, 25.2%), or on news and current affairs programs (*n* = 15, 13.5%). When asked about the timing of advertising, most young people recalled gambling advertising in the early evening before 8:30 pm (*n* = 75, 67.6%), followed by late evening after 8:30 pm (*n* = 50, 45.0%), afternoons (*n* = 26, 23.4%), and mornings (*n* = 10, 9.0%).

In relation to social media, over half of young people described seeing gambling advertisements on social media (*n* = 61, 55.0%). Just over a third of young people (*n* = 40, 36.0%) said that they saw gambling advertising on YouTube, for example before watching basketball videos:

I’ve seen them heaps in YouTube basketball videos. The ads pop up and they are the same as on the television. (12-year-old boy)

Others described seeing gambling advertising on YouTube “before I watch the highlights” of sporting matches, or in other non-gambling videos (for example gaming or YouTuber videos). A small number of young people described having to watch these advertisements before they could watch a YouTube video. For example, one girl aged 12 told researchers “sometimes you can skip ads, sometimes you have to watch it all”. Several described the specific content within these advertisements:

[I see them] on YouTube before I watch a video. A funny Sportsbet skit comes on. It’s not about gambling though... I see them when I watch highlights too. (15-year-old boy)

Fourteen (12.6%) young people said that they saw gambling advertisements on Instagram, including sponsored advertisements while they were scrolling on their news feed, or through sponsored posts. Five said they saw gambling advertisements on Snapchat, including one 12-year-old girl who stated “When you are looking through your friends’ stories, they pop up”*.*

Half (*n* = 59, 53.1%) recalled seeing a gambling advertisement specifically during their viewing of basketball, with a quarter (*n* = 27, 24.3%) recalling specific brands they had seen during their basketball viewing. Over one in ten (*n* = 15, 13.5%) recalled seeing gambling advertising during televised commercial breaks while watching basketball, with ten providing specific details about the advertisements they had seen:

[the advertisement says] choose your favourite player if you bet on the player to get MVP [most valuable player] and you win money. (14-year-old girl)

Ten (9.0%) recalled seeing gambling advertising at basketball stadiums, including gambling brand logos on the court, at half time on big screens, and around the stadiums. A few specifically recalled promotions for the company Ladbrokes during NBL matches. For example, one boy said:

[I see advertisements] sometimes in the break and I’ve seen the big Ladbrokes sign on the court in the Melbourne United games. (11-year-old boy)

### Impact of gambling advertising restrictions

The majority of young people stated that they continued to watch sport after 8:30 pm (*n* = 93, 83.7%). Some commented that they would “watch the whole game” particularly if it was their team playing or if it was “a close margin” within the game. Those who did not watch sport after 8:30 pm (*n* = 16, 14.4%) were younger participants who said this was due to their early bedtime, or older participants who said they did not watch much televised sport.

The majority of young people (*n* = 88, 79.3%) stated that there were too many gambling advertisements in sport and said there should be “none” or “less” advertisements. Some believed that gambling advertisements had a negative impact or were risky for young people, stating that gambling advertising within sport would teach young people that “they can’t enjoy sport without betting” or that betting on sport was “normal”. One fifth (*n* = 23, 20.7%) stated that they thought the amount of gambling advertisements did not need to change or they were unsure about the impact on young people. There were some young people who had not noticed gambling advertising, with one young person noting that she perceived that gambling advertisements would not affect her age group:

I haven’t really noticed them… I don’t think it would impact my age, but it may impact older kids, like 15 or 16. (13-year-old girl)

There were also young people who commented that they thought the amount of gambling advertising was appropriate. For example, the following 11-year-old boy stated that advertising was “slightly irritating because you want to watch sport, but I understand why [sport has gambling advertising] and don’t think there’s too much”. Another 12-year-old girl said that she disagreed there were too many gambling advertisements, highlighting that people were able to make choices about engaging in gambling:

Everyone has their own opinion and companies have to advertise to make money. But people can choose whether they want to gamble or not*.* (12-year-old girl)

Three quarters of young people agreed that sporting codes should do more to prevent young people from being exposed to advertising for gambling in sport (*n* = 84, 75.7%). Young people had a range of messages for sports organisations about their role in the promotion of gambling advertising, including that codes should be involved in reducing or banning gambling advertisements (*n* = 36, 32.1%):

People want to watch the game and not see the ads, they don’t need to be encouraged to have a bet or see the offers. (11-year-old boy)

A quarter (*n* = 29, 25.9%) said that sporting codes should think more about the impact gambling advertising has on young people, including the negative impacts of gambling (*n* = 14, 12.5%). Some said that sporting codes needed to do more to stand up against gambling advertising and that “codes should say something” (*n* = 16, 14.3%). For example, a few commented that individuals look up to athletes and that sporting codes should remember that they were “more about the sport not the gambling”. Others perceived that sporting codes had a responsibility to help young people understand the risks posed by gambling:

They should talk about the negative influences it has on our society to kids. They can really make a big difference with their impact and view in society*.* (14-year-old boy)

## Discussion

This paper provides enhanced understanding of young people’s exposure to, and awareness of, gambling advertising across media platforms since implementation of new gambling advertising restrictions in Australia in March 2018. There were five areas of discussion guided by the research questions.

First, this study contributes to existing research that has shown that young people are exposed to gambling advertising across a range of different media platforms. Consistent with other research, over 90% of young people reported seeing gambling advertising on television [23]. However, a new finding is that 55% of young people recalled seeing gambling advertising on social media platforms. While regulations have predominantly focused on traditional media platforms such as television, there are no regulations in Australia or elsewhere which restrict gambling advertising on social media platforms such as YouTube, Instagram, Snapchat, and Facebook. In the UK, there have been some attempts at enforcing restrictions on gambling advertisements online, with gambling advertisements appealing to young people banned on websites [55]. However, there have been no comprehensive regulations that target “below the line” marketing on social media platforms. As there has been very little research into young people’s exposure to gambling advertisements on social media, it is important to look to other areas of public health such as tobacco and alcohol, which have demonstrated that social media is an influential marketing space for companies that are restricted from traditional advertising strategies [33, 56]. While recommendations from the Australian government are that only young people aged 13 years and over should be able to create social media accounts [52], it is clear from this study that those as young as 11 and 12 are creating social media accounts or accessing social media sites. Social media may pose new challenges as parents may be unaware of the advertisements on these platforms, which limits discussions that may take place with young people about marketing they see. It is important that regulations keep pace with advances in technology to ensure that social media platforms fall under the same regulatory frameworks as traditional advertising channels.

Second, young people indicated that they see gambling advertising at all times of the day, but particularly in the early evening before 8:30 pm. This is an important finding as there may be a misperception that advertising restrictions in live sport also apply to other content before 8:30 pm. There are two gaps in Australian regulations that allow young people to be exposed to gambling advertisements prior to 8:30 pm. First, current regulations ban gambling advertising during “G” classified programs between 6:00 am–8:30 am and 4:00 pm–7:00 pm, or any television program directly targeting children from 5:00 am to 8:30 pm. However, young people identified seeing gambling advertisements during entertainment and reality style programs which may not be traditionally classified as directly targeting young people, but are promoted as “family friendly” viewing. Second, there are exemptions that allow gambling advertisements during news, current affairs, and sporting programs (until the recent amendments in relation to live sport). These programs typically are shown from 4:00 pm to 7:30 pm. As young people can recall gambling advertising in a range of sporting and non-sporting programs, and during times when advertising regulations are implemented, it is clear from this study that these loopholes within regulations need to be closed.

Third, many young people said that they continue watching sport after 8:30 pm (when bans on advertising in live sport cease to operate), and have indicated seeing gambling advertising specifically while watching sport. In this context, it is naïve to think that young people who are interested in watching sporting matches will turn off the television on a Friday, or Saturday night at 8:30 pm. This study demonstrates that sport continues to be a large contributor to young people’s exposure to gambling advertising. It also demonstrates the importance of creating regulations that are based on evidence relating to young people’s media viewing behaviours. Given that young people specifically stated that they continue to watch games which are played through the 8:30 pm cut-off, whistle to whistle bans are clearly necessary if their exposure to gambling advertising is to be limited. These bans should also include stadium-based advertising that may be visible to young people when watching live sport. Finally, we would argue that key exemptions to “low” audience channels create unintended loopholes which contradict the Government’s intentions of implementing regulations that aim to limit exposure to gambling advertising in sport for young people [43, 45].

Fourth, an important aspect of this research was the opportunity for young people to share their opinions on what could be done about exposure to gambling advertising. As in other studies [2], most young people thought that sporting codes should do more to protect them from exposure to gambling advertisements. While it is reassuring that some athletes and sporting teams have been supportive of reducing sponsorship relationships and revenue received from gambling companies [57, 58], this is still a difficult issue for athletes and teams to navigate when major sporting codes accept large advertising and sponsorship deals [59, 60]. However, the message from young people in this study about the need to remove gambling advertising from sport is clear.

Finally, these findings should help to set the research agenda outside Australia where knowledge about how young people understand and act on gambling advertising is limited. If policy makers are reluctant to act on data gathered in different jurisdictions or on “logic based on parallel evidence” ([61], p. 5), then it falls to researchers elsewhere to repeat experiments using similar methods, suitably adapted to local conditions, in order to avoid the costs of methodological novelty and to make data cumulative. Policy makers should not delay action on the basis that further research is needed, but rather should implement appropriate interventions on the basis of robust findings and sensible extrapolations, as they have done in many other areas in public health [62].

The study had a number of limitations. First, in the absence of substantial funding to evaluate the impact of new gambling advertising regulations in Australia, this study was funded via a small research support account held by ST. This restricted the number of interviews we could complete and restricted the study to three geographic regions of Victoria. However, many young people travel across the state for basketball games, and the study interviewed young people from a diverse range of geographic areas. Second, while the study aimed for diversity in the sample, the sample was skewed towards boys, and younger children, and was not representative of young people from different ethnic backgrounds. The *χ*^2^-tests of independence may also be biased given availability of data from a non-random sample. Finally, there may have been some social desirability bias in answering the questions in this study. While young people were told there were no right or wrong answers, they were aware that the research was examining the impact of gambling advertising on young people. This may have led to answers which were more critical of gambling advertising in sport, and more supportive of advertising restrictions.

## Conclusion

Young people are heavily exposed to gambling advertising and promotion across a wide range of media platforms, including social media, and at all times. The current regulatory systems fail to protect them from gambling promotions through sport and celebrity associations, and offer loopholes that enable such forms of promotion to thrive. Young people themselves are aware and critical of the ubiquity, intent and impact of gambling promotion, and the extent and nature of their own exposure with insights that should be noted by those responsible for their protection from such exposure. While aware that gambling promotion brings financial benefits to both sports and media organisations, young people believe that more should be done to protect them from this form of promotion which normalises gambling behaviour from early ages. There is now a clear body of evidence confirming that the current regulatory systems for gambling advertising are ineffective. While further research can and should investigate this and related issues, policy makers and sporting authorities that fail to act could be enabling the exposure of young people to forms of promotion that may encourage their interest and involvement in gambling.

## Abbreviations

AFL: Australian Football League
NBA: American National Basketball Association
NBL: Australian National Basketball League
SEIFA: Socio-Economic Indexes for Areas
SPSS: Statistical Package for the Social Sciences
UK: United Kingdom

## Footnotes

1 GambleAware is a charity funded by voluntary contributions from the gambling industry which was, until 2016, chaired by a senior figure from the gambling industry [41].

2 For researchers interested in replicating this study, young people were able to select from five different coloured drink bottles – clear, grey, red, green, and orange. The drink bottles were a very successful token of appreciation, and when set out on tables it encouraged young people and parents to inquire about what the research team was doing. As a note, red and green drink bottles were very popular.
